# Developing quality assurance tests for simultaneous Positron Emission Tomography – Magnetic Resonance imaging for radiotherapy planning

**DOI:** 10.1016/j.phro.2022.03.003

**Published:** 2022-04-20

**Authors:** Jonathan J. Wyatt, Hazel M. McCallum, Ross J. Maxwell

**Affiliations:** a Translational and Clinical Research Institute, Newcastle University, Newcastle, UK; b Northern Centre for Cancer Care, Newcastle upon Tyne Hospitals NHS Foundation Trust, Newcastle, UK

**Keywords:** Positron Emission Tomography, Magnetic resonance imaging, PET-MR, Quality Assurance, QA, Radiotherapy

## Abstract

**Background and purpose** Simultaneous Positron Emission Tomography – Magnetic Resonance (PET-MR) imaging can potentially improve radiotherapy by enabling more accurate tumour delineation and dose painting. The use of PET-MR imaging for radiotherapy planning requires a comprehensive Quality Assurance (QA) programme to be developed. This study aimed to develop the QA tests required and assess their repeatability and stability. **Materials and methods** QA tests were developed for: MR image quality, MR geometric accuracy, electromechanical accuracy, PET-MR alignment accuracy, Diffusion Weighted (DW)-MR Apparent Diffusion Coefficient (ADC) accuracy and PET Standard Uptake Value (SUV) accuracy. Each test used a dedicated phantom and was analysed automatically or semi-automatically, with in–house software. Repeatability was evaluated by three same-day measurements with independent phantom positions. Stability was assessed through 12 monthly measurements. **Results** The repeatability Standard Deviations (SDs) of distortion for the MR geometric accuracy test were ⩽0.7mm. The repeatability SDs in ADC difference from reference were ⩽3% for the DW-MR accuracy test. The PET SUV difference from reference repeatability SD was 0.3%. The stability SDs agreed within 0.6mm, 1 percentage point and 1.4 percentage points of the repeatability SDs for the geometric, ADC and SUV accuracy tests respectively. There were no monthly trends apparent. These results were representative of the other tests. **Conclusions** QA Tests for radiotherapy planning PET-MR have been developed. The tests appeared repeatable and stable over a 12-month period. The developed QA tests could form the basis of a QA programme that enables high-quality, robust PET-MR imaging for radiotherapy planning.

## 1 Introduction

Simultaneous Positron Emission Tomography – Magnetic Resonance (PET-MR) scanners enable acquiring both MR and PET functional information with high spatial alignment [1]. This has the potential to improve tumour delineation and to enable the metabolically active tumour sub-volumes to be identified with high accuracy through utilising the complementary information from both modalities [2]. Accurate delineation of these sub-volumes can enable dose painting strategies [3], potentially improving tumour control without increasing toxicities [4], [5]. A recent review found prostate boost volume delineation using Prostate Specific Membrane Antigen-PET, T2-weighted-MR and Diffusion Weighted (DW)-MR significantly improves the potential tumour control probability [6]. In addition quantitative PET-MR metrics, such as PET Standard Uptake Value (SUV) and DW-MR Apparent Diffusion Coefficient (ADC), have shown potential as imaging biomakers for treatment prognosis and response monitoring [7]. Several studies have demonstrated the clinical feasibility of using PET-MR in the radiotherapy position for different treatment sites [8], [9], [10].

To use PET-MR imaging for radiotherapy planning, a comprehensive PET-MR Quality Assurance (QA) programme needs to be developed. There are currently consensus guidelines on PET-MR QA for the diagnostic setting [11]. However, images used for radiotherapy planning must meet additional requirements compared to diagnostic imaging [12]. These include high geometric accuracy over the entire field of view [13], sufficient image quality for accurate delineation of tumour and organ at risk boundaries [14], a high degree of spatial alignment between images acquired in the same session [15] and high mechanical accuracy in couch and laser movements to ensure reproducible patient positioning [16]. In addition if quantitative functional information is being used for automatic image segmentation or treatment response monitoring, then the accuracy and stability of these quantitative metrics needs to be assured [17], [18]. Diffusion Weighted (DW)-MR is the most investigated functional MR technique for radiotherapy planning [19] and so a test of ADC accuracy is likely to be necessary. If other functional MR techniques are being used for radiotherapy planning (eg Dynamic Contrast Enhanced MR) then additional tests would also be required. Therefore a radiotherapy dedicated PET-MR QA programme needs to be developed. This would need to include the current recommendations for radiotherapy MR imaging: MR image quality, MR geometric accuracy and electromechanical accuracy tests, as well as PET-MR specific tests covering PET-MR alignment accuracy, DW-MR ADC accuracy and PET SUV accuracy.

Radiotherapy adapted PET-MR systems have been evalauted using QA phantoms. PET and MR image quality has been evaluated in a head and neck radiotherapy setup using uniform PET and MR phantoms [20] and in a pelvic radiotherapy setup using image quality phantoms [21]. Methods to correct the PET attenuation from the radiotherapy setup [22], [21] have also been evaluated. However, to the best of the author’s knowledge, a comprehensive set of tests for a routine QA programme have not been evaluated in the literature previously. Therefore the aim of this study was to develop the tests needed for such a programme and to assess the repeatability of these tests and their stability over a 12 month period.

## 2 Materials and methods

**Fig. 1:**
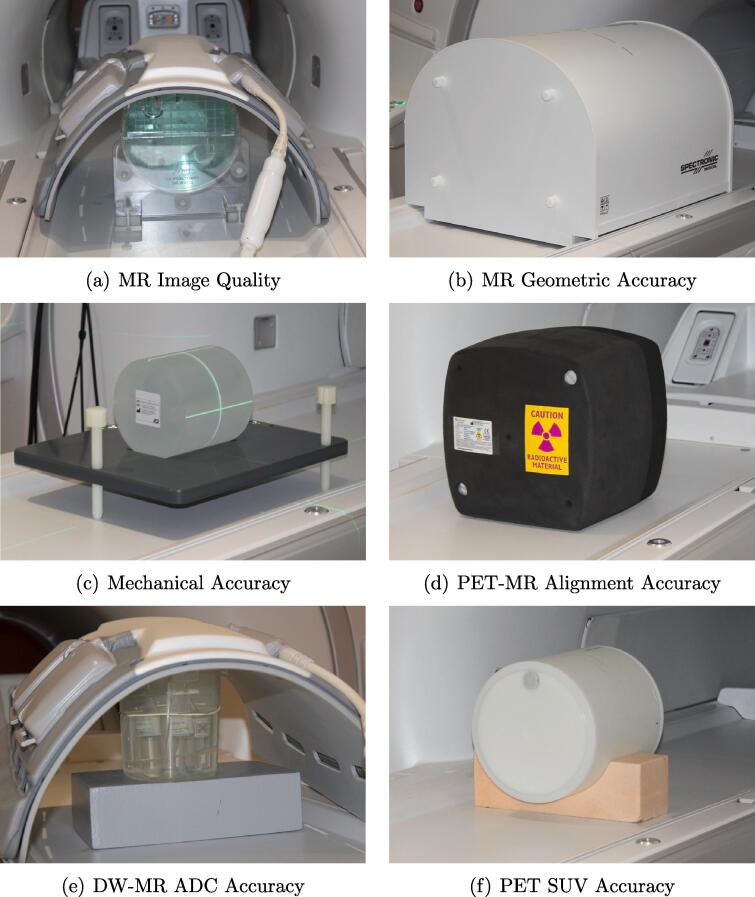
Photographs of the phantoms and setup used for each of the six QA tests evaluated. Each test was carried out three times on the same day using independent phantom setups (repeatability measurements) and once a month for 12 months (stability measurements). Images were acquired using the spine coil and anterior array coil for MR image quality and DW-MR ADC accuracy tests, and the in-built body coil/PET detector for the other tests.

PET-MR radiotherapy QA tests were developed for MR image quality, MR geometric accuracy, electromechanical accuracy, PET-MR alignment accuracy, PET SUV accuracy and DW-MR ADC accuracy (Fig. 1). Repeatability was determined using three independent same-day measurements and stability through monthly measurements from October 2020 to September 2021, all acquired on a Signa 3T PET-MR scanner (version MP26, GE Healthcare, Milwaukee, USA) by the same observer. Measurements took approximately two hours with a further 15 min for image analysis. Repeatability and stability were assessed by the Standard Deviation (SD).

### 2.1 MR image quality

MR image quality was assessed with the American College of Radiologists (ACR) large image quality phantom [23]. Images were acquired using the in-built spine coil and anterior array coil used for pelvic imaging. Images were not acquired with the radiotherapy couch and coil bridge.

The recommended T1-weighted (ACR T1) and double-echo T2-weighted (ACR T2) axial spin echo sequences were acquired. Images were analysed according to the ACR recommendations using in–house developed Matlab software based upon open source software [24] and substantially modified to make it more accurate and robust. High-contrast resolution was assessed as the smallest diameter line of holes detectable in a horizontal or vertical array. Slice thickness accuracy was determined by imaged profile of two angled ramps. Slice position accuracy used crossed 45° wedges at either end of the phantom. The image uniformity test was the ratio of near-minimum and near-maximum pixel values in the uniform phantom compartment. The ghosting ratio was the ratio of pixel values outside the phantom to those within the uniform compartment. The low-contrast object detection was the total number of visible ‘spokes’ of disks with decreasing contrast (5.1%-1.4%) and diameter (7.0-1.5mm). This test was performed manually by a single observer using RadiAnt DICOM Viewer (version 4.6.9.18463, Medixant, Posnan, Poland). The geometric accuracy ACR test was omitted due to the dedicated geometric accuracy test.

### 2.2 MR geometric accuracy

MR Geometric accuracy was assessed using the large field of view GRADE phantom (Spectronic Medical, Helsingborg, Sweden). This consisted of ∼1,200 spherical markers embedded in expanded foam in a grid pattern. Images were acquired with recommended 2D and 3D sequences and automatically analysed using the vendor provided software (version 1.0.46) which calculated the distortion shift of each marker as the absolute Euclidian distance in 3D from the marker position in the image to the known reference position [25].

The markers were grouped in concentric circles at increasing distances from the isocentre and the mean distortion in each group calculated. Repeatability was assessed by then calculating the mean and SD of those group means over the three repeats. In addition, each marker was uniquely identified and so the mean and SD of distortion for each marker over the three repeats was calculated (${\overset{‾}{D}}_{n}\pm \sigma_{n}$ for marker *n*). A few markers on the periphery of the phantom were not identified in all three repeatability measurements and were excluded from the analysis (25/785 and 16/852 markers for the 2D and 3D sequences respectively). The mean SD of all markers over the three repeats was calculated using [25]

(1) $$\overset{‾}{\sigma }=\frac{1}{N}\overset{N}{\underset{n=1}{\sum }}\sigma_{n},$$

where $\sigma_{n}$ was the standard deviation of the $n^{\mathrm{th}}$ marker and *N* was the number of markers common to all images over the three repeats. The range in distortion for each marker over the three repeats was also calculated and the mean range over all markers determined. Monthly stability was assessed using the same methods. Similarly, markers not common to all 12 monthly measurements were excluded in the SD and range of distortion calculations (26 for the 2D and 23 for the 3D).

### 2.3 Mechanical accuracy

The Aquarius phantom (LAP GmbH, Schwarzenbruck, Germany) was used to measure the alignment of the internal and external lasers with the scanner imaging plane. The phantom contained 10cm long cross-planes in the transverse, sagittal and coronal axes in a copper sulphate solution. The phantom was aligned to the external lasers in all three planes, then aligned in the superior-inferior direction on the internal laser and shifted to isocentre. A 3D fast spin echo sequence centred on the scanner isocentre was acquired. The centre of the cross-planes and points ±5cm in each axis were manually marked on the image in RayStation (v9b, RaySearch Laboratories, Stockholm, Sweden) and the transverse and rotational offsets in each plane from the image centre calculated using an in–house script.

The coincidence of the two lateral external lasers was assessed across the scanner couch using hand shielding. External laser movements were assessed by moving the relevant laser by a set amount and measuring the distance traversed with a ruler. The coincidence of the sagittal internal and external lasers was measured using a ruler. The electromechanical accuracy of the couch movements was assessed using a ruler fixed to the scanner couch aligned with the external sagittal laser. For both external laser and couch motions the final shift was back to the zero position to assess hysteresis.

### 2.4 PET-MR alignment accuracy

PET-MR alignment was assessed using the VQC phantom (GE Healthcare) containing five solid low activity (∼0.7MBq) ^68^Ge spheres in a geometric grid pattern, with each sphere having one MR-visible sphere superior and inferior of it. The phantom is designed so that the halfway point between each MR sphere pair should exactly coincide wih the PET sphere. MR and PET images were acquired and analysed within RaySation using an in–house script which automatically identified all the PET and MR spheres. For each pair of MR spheres the point halfway between the two sphere centroids was calculated. A six-degrees-of-freedom rigid registration was performed to align these calculated MR points with the measured PET sphere centroids, giving a measure of PET-MR alignment.

### 2.5 DW-MR apparent diffusion coefficient accuracy

DW-MR ADC accuracy was assessed using an in–house phantom, consisting of three sealed vials containing 100ml of n-nonane, n-undecane and tridecane respectively, surrounded by ∼800ml of water. Images were acquired using the anterior array coil and spine coil. The phantom temperature was allowed to equilibrate with the room temperature and measured before and after image acquisition. A single-shot Echo Planar Imaging DW-MR sequence was acquired with a single coronal slice through the vials using b-values $50\;s\;{\mathrm{mm}}^{-2}$ and $800\;s\;{\mathrm{mm}}^{-2}$, with two averages for $b=800\;s\;{\mathrm{mm}}^{-2}$ to improve Signal to Noise Ratio (SNR). Initial measurements with 8 b-values ($50\;s\;{\mathrm{mm}}^{-2}$-$1500\;s\;{\mathrm{mm}}^{-2}$) showed that the natural logarithm of signal intensity was highly linear with b-value, as expected for a free diffusion phantom [26]. Therefore only two b-values were acquired for speed.

The images were analysed in Medical Interactive Creative Environment Toolkit (version 1.0.8, Umea University, Sweden) [27]. Each vial was automatically segmented on the $b=50\;s\;{\mathrm{mm}}^{-2}$ image and mean ADC calculated. Reference ADC values were determined from literature values as a function of temperature (values given in supplementary material) [28], and linearly interpolated from the measured phantom temperature. The percentage difference in ADC between measured and reference was calculated.

### 2.6 PET standard uptake value accuracy

A uniform PET activity phantom was used to assess PET SUV accuracy containing ∼30MBq of ^18^F-Fluorodeoxyglucose (FDG). A 10 min single bed-position PET scan was acquired without the radiotherapy couch top, coil bridge and anterior array coil. The PET image was reconstructed using an Ordered Subset Expectation Maximum reconstruction with 16 subsets and 4 iterations and a 5.0mm Gaussian filter with resolution recovery and time of flight information.

Repeatability measurements were acquired by adding additional activity between scan 1 & 2 and scan 2 & 3 so that each acquisition had a unique activity fill (more details in supplementary material). Phantom attenuation correction was based on a CT image of the phantom which was rigidly registered to the PET image, visually assessed and then combined with an attenuation coefficient map of the spine coil and PET-MR couch for the final PET image reconstruction (see supplementary material). Images were automatically analysed using an in–house script within RayStation which placed a 18cm diameter and 18cm long cylindrical ROI at the phantom centre. The mean SUV within the ROI was calculated and the percentage difference to the reference SUV determined.

## 3 Results

**Fig. 2:**
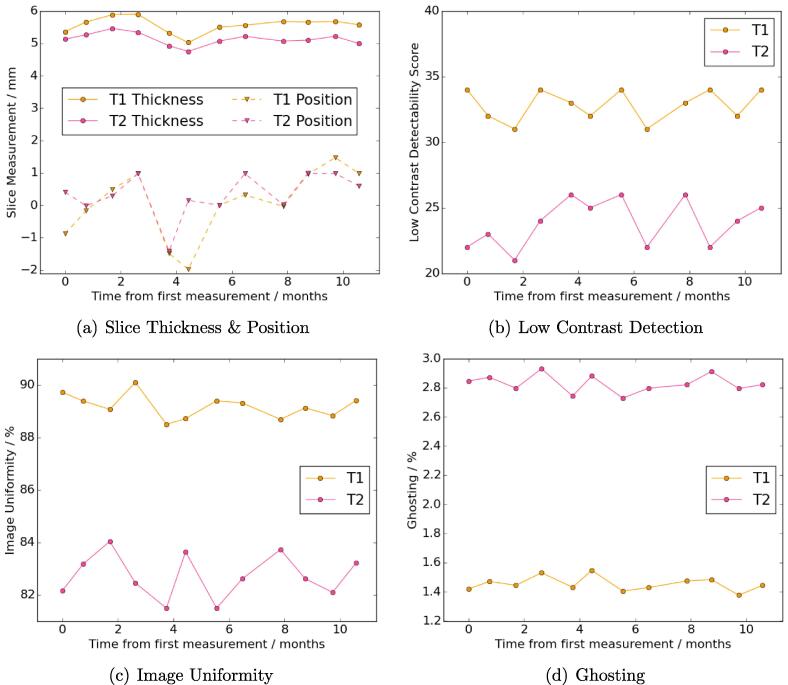
Monthly stability plots of the MR image quality test. The plots show slice thickness and position (a), low contrast detection (b), image uniformity (c) and image ghosting (d) measurements.

Example images of all tests are shown in supplementary material (Figures S1-S6). All the MR image quality repeatability and stability measurements were within the recommended tolerance values except for the low-contrast detectability scores (Table 1) and there were no monthly trends (Fig. 2, the spatial resolution results are not shown since every month was identical). The monthly stability means and SDs were very similar to the repeatability means and SDs for all tests except slice position, ghosting and image uniformity (T2).

**Table 1:** The repeatability and stability results for all tests. T1/T2 refers to the ACR T1/T2 image series respectively. 2D/3D refers to the 2D and 3D geometric accuracy sequences respectively. d indicates distance from the scanner isocentre. EL refers to the external lasers mounted on the laser bridge and IL to the scanner internal lasers.The reference column indicates the tolerance values from the ACR manual for the MR image quality test, the recommended tolerance for geometric distortion in MR for radiotherapy and the reference values for the other tests.

**Test**	**Component**	**Reference**	**Repeatability**		**Stability**	
			*Mean*	*SD*	*Mean*	*SD*

1) MR Image Quality	Spatial Resolution (T1) [mm]	≤1.0	1.0	0.0	1.0	0.0
	Spatial Resolution (T2) [mm]	≤1.0	1.0	0.0	1.0	0.0
	Slice Thickness (T1) [mm]	5±0.7	5.3	0.2	5.6	0.2
	Slice Thickness (T2) [mm]	5±0.7	4.9	0.2	5.1	0.2
	Slice Position (T1) [mm]	⩽5	1.3	0.6	0.1	1.1
	Slice Position (T2) [mm]	⩽5	1.4	0.5	0.3	0.7
	Image Uniformity (T1) [%]	⩾82	88.8	0.3	89.1	0.5
	Image Uniformity (T2) [%]	⩾82	85.0	0.1	82.7	0.8
	Ghosting (T1) [%]	⩽3	0.7	0.05	1.5	0.05
	Ghosting (T2) [%]	⩽3	0.9	0.04	2.8	0.06
	Low-Contrast Detection (T1)	⩾37	35	1	33	1
	Low-Contrast Detection (T2)	⩾37	26	1	24	2
						
2) MR Geometric Accuracy	SD of Distortion (2D) [mm]	–	0.4	–	0.3	–
	SD of Distortion (3D)[mm]	–	0.2	–	0.3	–
	Range of Distortion (2D) [mm]	–	0.7	1.5	0.9	0.3
	Range of Distortion (3D) [mm]	–	0.4	0.2	0.9	0.3
	Distortion d<10cm (2D) [mm]	⩽2.0	0.27	0.07	0.27	0.05
	Distortion 10⩽d<15cm (2D)[mm]	⩽2.0	0.44	0.02	0.40	0.05
	Distortion 15⩽d<20cm (2D) [mm]	⩽2.0	0.82	0.04	0.74	0.05
	Distortion 20⩽d<25cm (2D) [mm]	⩽2.0	2.2	0.2	1.90	0.04
	Distortion d⩾25cm (2D) [mm]	–	7.5	0.7	4.7	0.1
	Distortion d<10cm (3D) [mm]	⩽2.0	0.23	0.03	0.27	0.04
	Distortion 10⩽d<15cm (3D) [mm]	⩽2.0	0.33	0.02	0.34	0.05
	Distortion 15⩽d<20cm (3D) [mm]	⩽2.0	0.60	0.02	0.62	0.05
	Distortion 20⩽d<25cm (3D) [mm]	⩽2.0	1.63	0.05	1.73	0.05
	Distortion d⩾25cm (3D) [mm]	–	5.99	0.03	4.85	0.04
						
3) Mechanical Accuracy	EL Right-Left Offset [mm]	0.0	0.1	0.3	0.3	0.4
	EL Ant-Post Offset [mm]	0.0	0.0	0.3	0.4	0.1
	EL Pitch Angle [^o^]	0.0	-0.1	0.1	0.0	0.2
	EL Roll Angle [^o^]	0.0	0.2	0.2	0.0	0.3
	EL Yaw Angle [^o^]	0.0	-0.1	0.1	-0.1	0.2
	EL Lateral Coincidence [mm]	0.0	0.0	0.0	0.0	0.0
	EL Movements [mm]	0.0	0.0	0.1	0.0	0.1
	EL-IL Right-Left Difference [mm]	0.0	1.7	0.3	1.8	0.2
	IL Sup-Inf Offset [mm]	0.0	2.2	0.6	1.6	0.6
	Couch Movements [mm]	0.0	0.1	0.5	0.2	0.5
						
4) PET-MR Alignment	Right-Left Difference [mm]	0.0	0.15	0.03	-0.2	0.1
	Ant-Post Difference [mm]	0.0	0.12	0.02	0.0	0.1
	Sup-Inf Difference [mm]	0.0	0.02	0.07	0.2	0.1
	Pitch Angle [^o^]	0.0	0.13	0.07	-0.02	0.05
	Roll Angle [^o^]	0.0	-0.01	0.00	0.00	0.03
	Yaw Angle [^o^]	0.0	0.01	0.05	-0.07	0.07
						
5) DW-MR ADC Accuracy	Nonane Difference [%]	0	2	1	3	1
	Undecane Difference [%]	0	-2	2	0	1
	Tridecane Difference [%]	0	-2	3	0	2
						
6) PET SUV Accuracy	SUV Difference [%]	0.0	1.3	0.5	2.1	1.9

**Fig. 3:**
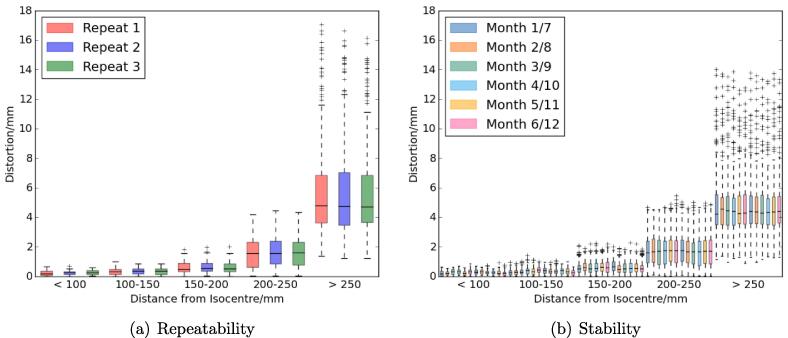
Boxplots of the distributions of distortions at different distances from the isocentre for the 3D sequence for the repeatability (a) and monthly stability (b) measurements. The repeat and monthly measurements are displayed in the order acquired and shown as different colours. For the monthly measurements each colour represents two months, six months apart. The 2D sequence results showed very similar pattern.

The MR geometric accuracy test had similar distributions of distortions at different distances from the isocentre between repeats and between months (Fig. 3). The mean monthly SDs of distortion were within 0.1mm of the repeatability values (Table 1).

**Fig. 4:**
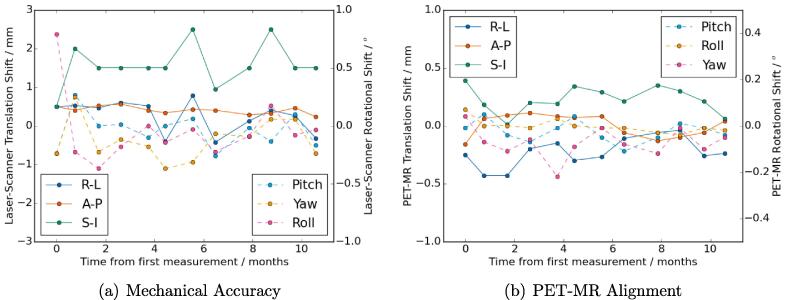
Monthly stability plot of mechanical accuracy (a) and PET-MR alignment (b) measurements. Both plots show translational (left axis, solid lines) and rotational (right axis, dashed lines) differences.

The mechanical accuracy stability means agreed within one stability SD with the repeatability means (except for the external laser anterior-posterior offset, which agreed within 0.4mm) and the stability SDs were within 0.2mm or 0.1° of the repeatability SDs (Table 1). There were no trends in the monthly measurements for the mechanical accuracy or PET-MR alignment tests (Fig. 4).

**Fig. 5:**
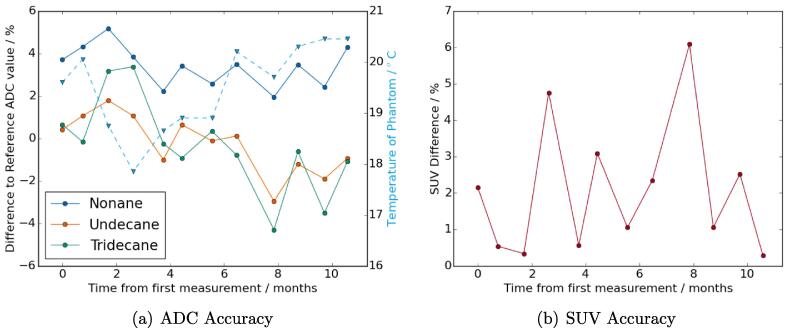
Monthly stability plot of percentage difference in mean ADC value to temperature-corrected literature reference value for each vial (a) and plot of percentage difference in mean SUV within phantom to reference activity (b). Plot (a) also shows measured temperature of phantom (blue dashed line).

The DW-MR ADC stability measurements showed no monthly trends or trend with phantom temperature (Fig. 5). There were systematic discrepancies between the different alkanes, with the nonane vial always having a positive difference to the reference value and the undecane and tridecane vials being around zero difference. There was no monthly trend in the SUV accuracy measurements (Fig. 5), although all measured SUVs had a positive difference, suggesting a small systematic SUV over-estimation.

## 4 Discussion

This study has developed phantoms and analysis software for PET-MR QA tests for radiotherapy, covering MR image quality, MR geometric distortion, mechanical accuracy, PET-MR alignment, DW-MR ADC accuracy and PET SUV accuracy. The tests appeared repeatable, with repeatability SDs ⩽0.7mm for all differences to reference in distance, ⩽0.2° for angle differences, ⩽3% for percentage differences and 1 spoke for low-contrast detectability. The stability SDs were similar to repeatability, within 0.2mm,0.1°, 1 percentage point and 1 spoke, except for slice position and SUV accuracy. These were within 0.5mm and 1.5 percentage points respectively. There were also no monthly trends, suggesting the tests were stable.

The MR image quality tests had small repeatability SDs relative to the ACR tolerances and very similar monthly stability SDs, within 0.2mm,0.2 percentage points or 1 spoke, except for the T1 slice position and T2 image uniformity measurements. These were 0.5mm and 0.7 percentage points larger respectively. This suggests there is more variation in scanner performance for these parameters, although the absence of any trends (Fig. 2) implies that the performance is still stable. Adjeiwaah et al. investigated the repeatability of the ACR phantom using the same coil setup and scanner and reported repeatability SDs that agreed with the results here within 0.1mm (slice thickness), 0.9mm (slice position), 0.5 percentage points (image uniformity) and 0.07 percentage points (ghosting) [29]. They also reported high stability over 4 years in agreement with the results found here. Only the low-contrast detectability test was not within the ACR tolerance levels, likely due to using the spine and anterior array coils rather than the head and neck coil recommended by ACR. However this ensured that the coils used for radiotherapy imaging were being tested.

The MR geometric accuracy tests appeared highly repeatable and stable, with both the repeatability and stability mean SDs of distortion being ⩽0.4mm for 2D and 3D sequences. This is small compared to the 2mm acceptable limit of distortion for MR-only radiotherapy [30]. The test showed excellent agreement with a previous study using the same phantom on different MR scanners [25]. The stability SDs also agreed within 0.04mm of the SDs reported from five geometric distortion measurements over a 15 month period [31]. This suggests that the repeatability and stability of MR geometric accuracy test reported here is equivalent to those reported in the literature as suitable for clinical use.

The mechanical accuracy tests were highly repeatable and stable over time, with all SDs being ⩽0.6mm and all mean differences being within one SD of zero except two. These were small compared to the recommended tolerances of ±2mm [16]. The larger differences were the external-internal laser difference and the internal laser offset to the scanner isocentre. Given the high coincidence between the external laser and scanner isocentre (0.1±0.3mm) this indicates that the larger differences were due to the internal laser being misaligned by ∼2mm. This is within the scanner specification but confirms the requirement of external lasers for radiotherapy patient setup.

The PET-MR alignment accuracy test was highly repeatable, with repeatability SDs <0.1mm and <0.1°. The stability SDs were larger in most directions, but still very small (0.1mm and <0.1°) indicating a very stable system. There is not a recognised clinical tolerance since PET-MR is an emerging modality, however both SDs were significantly smaller than the typical PET voxel dimensions of 2–3mm. High PET-MR alignment was expected because the mechanical positions of the PET and MR imaging systems did not change during this study. However, given a major benefit of PET-MR scanners is the high intrinsic spatial alignment of the images, regular testing PET-MR alignment is important for providing assurance. To the best of the authors’ knowledge this has not been published in the literature, although it is recommended to regular assess PET-MR alignment for diagnostic PET-MR QA [11].

The DW-MR ADC accuracy test also demonstrated good repeatability, with SDs of differences ⩽3%. This compares well with values of other ADC free-diffusion phantoms. Chenevert et al. reported an ADC phantom consisting of distilled water in an ice-water bath was repeatable to within ±5% [32]. The stability measurements had similar SDs (agreeing within 1 percentage point with the repeatability SDs) and agreed with the repeatability measurements within one repeatability SD. This suggests the scanner performance was stable over the period measured. The vial containing nonane appeared to have a small but systematic bias. This may be due to the location of the nonane vial being further from the image centre than the other two vials, which can influence ADC measurements [33]. The stability results also show good agreement with similar studies. Winfield et al. reported coefficients of variation ⩽4% in ADC measurements over 20 months for a phantom with five sucrose solutions ranging from 0%-20% sucrose in an ice-water bath [34]. This suggests that the evaluated DW-MR ADC accuracy test was similarly repeatable and stable to other phantoms reported in the literature.

Finally the PET SUV accuracy test showed substantially larger monthly variation (SD 1.9%) to the repeatability SD (0.5%). This suggests variation in calibrator and scanner performance over time had a larger impact on SUV accuracy then the inherent uncertainty of the test measurement itself. There did also appear to be a bias in the results, with all differences being positive, suggesting the scanner systematically over-estimated the SUVs in the phantom. This may be due to small errors in the scanner applied attenuation correction maps of the PET-MR couch and MR receive coil, which are not present when the PET system undergoes quarterly calibration. However, this effect was small with all differences to the reference value being within 6% and 10/12 measurements being with 3%. This is consistent with studies indicating drifts in calibrator performance over time of approximately 4% [35]. One study investigating longitudinal changes in PET SUV measurements for six different scanners reported a large variability in practice, with changes in SUV differences ranging from 0.3% to 58.6% [36]. Half of the scanners reported changes in SUV differences greater than the 6% range measured here. This highlights the importance of regular PET SUV accuracy measurements to ensure high quality, quantitative PET images.

A potential limitation of this study was that the MR image quality and PET SUV accuracy tests were not acquired with the radiotherapy flat couch and coil bridge. This was done because the radiotherapy hardware causes a significant drop in MR SNR of 45% and PET activity of 17.7% [21]. This would have reduced the sensitivity of the tests to detect changes in MR/PET performance, which was their primary aim. However, not doing so did mean that the full clinical setup was not evaluated on a monthly basis. Additional QA tests using the radiotherapy hardware could be added, at the cost of additional QA time.

Another limitation was that only one scanner was evaluated. Future work will investigate the generalisability of these QA tests to scanners from other manufacturers in other centres. This data, combined with the repeatability and stability data reported here and considerations of the clinical impact of variations from ideal performance, will then be used to generate QA tolerances and test frequencies.

In conclusion, tests for a PET-MR radiotherapy QA programme have been developed. The tests appeared repeatable and stable over a 12-month period, although monthly variation was larger than test repeatability for PET SUV accuracy and two of the MR image quality tests. Future work will derive appropriate tolerance levels and test frequencies, which combined with these tests will form a comprehensive QA programme. This will enable high-quality, robust PET-MR imaging to be used for radiotherapy planning.

## Declaration of Competing Interest

The authors declare that they have no known competing financial interests or personal relationships that could have appeared to influence the work reported in this paper.

## References

[1] Monti S., Cavaliere C., Covello M., Nicolai E., Salvatore M., Aiello M. (2017). An Evaluation of the Benefits of Simultaneous Acquisition on PET/MR Coregistration in Head/Neck Imaging. J Healthc Eng.

[2] Thorwarth D., Leibfarth S., Mönnich D. (2013). Potential role of PET/MRI in radiotherapy treatment planning. Clin Transl Imaging.

[3] van der Heide U.A., Houweling A.C., Groenendaal G., Beets-Tan R.G., Lambin P. (2012). Functional MRI for radiotherapy dose painting. Magn Reson Imaging.

[4] Galvin J.M., De Neve W. (2007). Intensity modulating and other radiation therapy devices for dose painting. J Clin Oncol.

[5] von Eyben F.E., Kiljunen T., Kangasmaki A., Kairemo K., von Eyben R., Joensuu T. (2016). Radiotherapy Boost for the Dominant Intraprostatic Cancer Lesion—A Systematic Review and Meta-Analysis. Clin Genitourin Cancer.

[6] Zamboglou C., Eiber M., Fassbender T.R., Eder M., Kirste S., Bock M. (2018). Multimodal imaging for radiation therapy planning in patients with primary prostate cancer. Phys Imaging in Radiat Oncol.

[7] Daniel M., Andrzejewski P., Sturdza A., Majercakova K., Baltzer P., Pinker K. (2017). Impact of hybrid PET/MR technology on multiparametric imaging and treatment response assessment of cervix cancer. Radiother Oncol.

[8] Winter R.M., Leibfarth S., Schmidt H., Zwirner K., Mönnich D., Welz S. (2018). Assessment of image quality of a radiotherapy-specific hardware solution for PET/MRI in head and neck cancer patients. Radiother Oncol.

[9] Olin A.B., Hansen A.E., Rasmussen J.H., Ladefoged C.N., Berthelsen A.K., Håkansson K. (2020). Feasibility of Multiparametric Positron Emission Tomography/Magnetic Resonance Imaging as a One-Stop Shop for Radiation Therapy Planning for Patients with Head and Neck Cancer. Int J Radiat Oncol Biol Phys.

[10] Ahangari S., Hansen N.L., Olin A.B., Nøttrup T.J., Ryssel H., Berthelsen A.K. (2021). Toward PET/MRI as one-stop shop for radiotherapy planning in cervical cancer patients. Acta Oncol.

[11] Valladares A., Ahangari S., Beyer T., Boellaard R., Chalampalakis Z., Comtat C. (2019). Clinically Valuable Quality Control for PET/MRI Systems: Consensus Recommendation From the HYBRID Consortium. Front Phys.

[12] Paulson E.S., Erickson B., Schultz C., Li X.A. (2015). Comprehensive MRI simulation methodology using a dedicated MRI scanner in radiation oncology for external beam radiation treatment planning. Med Phys.

[13] Kapanen M., Collan J., Beule A., Seppälä T., Saarilahti K., Tenhunen M. (2013). Commissioning of MRI-only based treatment planning procedure for external beam radiotherapy of prostate. Magn Reson Med.

[14] Liney G.P., Moerland M.A. (2014). Magnetic resonance imaging acquisition techniques for radiotherapy planning. Semin Radiat Oncol.

[15] Thorwarth D., Beyer T., Boellaard R., Ruysscher D.D., Grgic A., Lee J.A. (2012). Integration of FDG- PET/CT into external beam radiation therapy planning. Nuklearmedizin.

[16] Mutic S, Palta JR, Butker EK, Das IJ, Huq MS, Loo LND, et al. Quality assurance for computed-tomography simulators and the computed-tomography-simulation process: Report of the AAPM Radiation Therapy Committee Task Group No. 66. Med Phys 2003;30:2762–2792. doi: 10.1118/1.1609271. 10.1118/1.1609271 14596315

[17] Xing L. (2008). Quality Assurance of Positron Emission Tomography/Computed Tomography for Radiation Therapy. Int J Radiat Oncol Biol Phys.

[18] Keenan K.E., Ainslie M., Barker A.J., Boss M.A., Cecil K.M., Charles C. (2018). Quantitative magnetic resonance imaging phantoms: A review and the need for a system phantom. Magn Reson Med.

[19] Olsson L.E., Johansson M., Zackrisson B., Blomqvist L.K. (2019). Basic concepts and applications of functional magnetic resonance imaging for radiotherapy of prostate cancer. Phys Imaging Radiat Oncol.

[20] Paulus D.H., Thorwath D., Schmidt H., Quick H.H. (2014). Towards integration of PET/MR hybrid imaging into radiation therapy treatment planning. Med Phys.

[21] Wyatt J.J., Howell E., Lohezic M., McCallum H.M., Maxwell R.J. (2021). Evaluating the image quality of combined positron emission tomography-magnetic resonance images acquired in the pelvic radiotherapy position. Phys Med Biol.

[22] Taeubert L., Berker Y., Beuthien-Baumann B., Hoffmann A.L., Troost E.G.C., Kachelrieß M. (2020;65:2302.). CT-based attenuation correction of whole-body radiotherapy treatment positioning devices in PET/MRI hybrid imaging. Phys Med Biol.

[23] American College of Radiology. Magnetic resonance imaging quality control manual. Technical report; 2015.

[24] Sun J., Barnes M., Dowling J., Menk F., Stanwell P., Greer P.B. (2015). An open source automatic quality assurance (OSAQA) tool for the ACR MRI phantom. Australas Phys Eng Sci Med.

[25] Wyatt J., Hedley S., Johnstone E., Speight R., Kelly C., Henry A. (2018). Evaluating the repeatability and set-up sensitivity of a large field of view distortion phantom and software for magnetic resonance-only radiotherapy. Phys Imaging Radiat Oncol.

[26] McHugh D.J., Zhou F.L., Wimpenny I., Poologasundarampillai G., Naish J.H., Cristinacce P.L.H. (2018). A biomimetic tumor tissue phantom for validating diffusion-weighted MRI measurements. Magn Reson Med.

[27] Nyholm T., Berglund M., Brynolfsson P., Jonsson J. (2015). EP-1533: ICE-Studio – An Interactive visual research tool for image analysis. Radiother Oncol.

[28] Tofts P., Lloyd D., Clark C., Barker G., Parker G., McConville P. (2000). Test liquids for quantitative MRI measurements of self-diffusion coefficient in vivo. Magn Reson Med.

[29] Adjeiwaah M., Garpebring A., Nyholm T. (2020). Sensitivity analysis of different quality assurance methods for magnetic resonance imaging in radiotherapy. Phys Imaging Radiat Oncol.

[30] Weygand J., Fuller C.D., Ibbott G.S., Mohamed A.S., Ding Y., Yang J. (2016). Spatial Precision in Magnetic Resonance Imaging-Guided Radiation Therapy: The Role of Geometric Distortion. Int J Radiat Oncol Biol Phys.

[31] Ranta I., Kemppainen R., Keyriläinen J., Suilamo S., Heikkinen S., Kapanen M. (2019). Quality assurance measurements of geometric accuracy for magnetic resonance imaging-based radiotherapy treatment planning. Phys Med.

[32] Chenevert T.L., Galbán C.J., Ivancevic M.K., Rohrer S.E., Londy F.J., Kwee T.C. (2011). Diffusion coefficient measurement using a temperature-controlled fluid for quality control in multicenter studies. J Magn Reson Imaging.

[33] Malyarenko D., Galbán C.J., Londy F.J., Meyer C.R., Johnson T.D., Rehemtulla A. (2013). Multi-system repeatability and reproducibility of apparent diffusion coefficient measurement using an ice-water phantom. J Magn Reson Imaging.

[34] Winfield J.M., Collins D.J., Priest A.N., Quest R.A., Glover A., Hunter S. (2016). A framework for optimization of diffusion-weighted MRI protocols for large field-of-view abdominal-pelvic imaging in multicenter studies. Med Phys.

[35] Lockhart C.M., MacDonald L.R., Alessio A.M., McDougald W.A., Doot R.K., Kinahan P.E. (2011). Quantifying and Reducing the Effect of Calibration Error on Variability of PET/CT Standardized Uptake Value Measurements. J Nucl Med.

[36] Doot R.K., Pierce L.A., Byrd D., Elston B., Allberg K.C., Kinahan P.E. (2014). Biases in Multicenter Longitudinal PET Standardized Uptake Value Measurements. Transl Oncol.

