# Supplementary material for: Developing quality assurance tests for simultaneous Positron Emission Tomography – Magnetic Resonance imaging for radiotherapy planning
Source: Phys Imaging Radiat Oncol. 2022 Apr 20;22:28–35. doi: 10.1016/j.phro.2022.03.003 (PMC9048159; doi:10.1016/j.phro.2022.03.003)
Supplement: Supplementary data 1 [file mmc1.pdf]

Supplementary Material A: Example Images

Example MR and PET images for each test are shown in figures S1 - S6. All images are taken from the first month of testing.

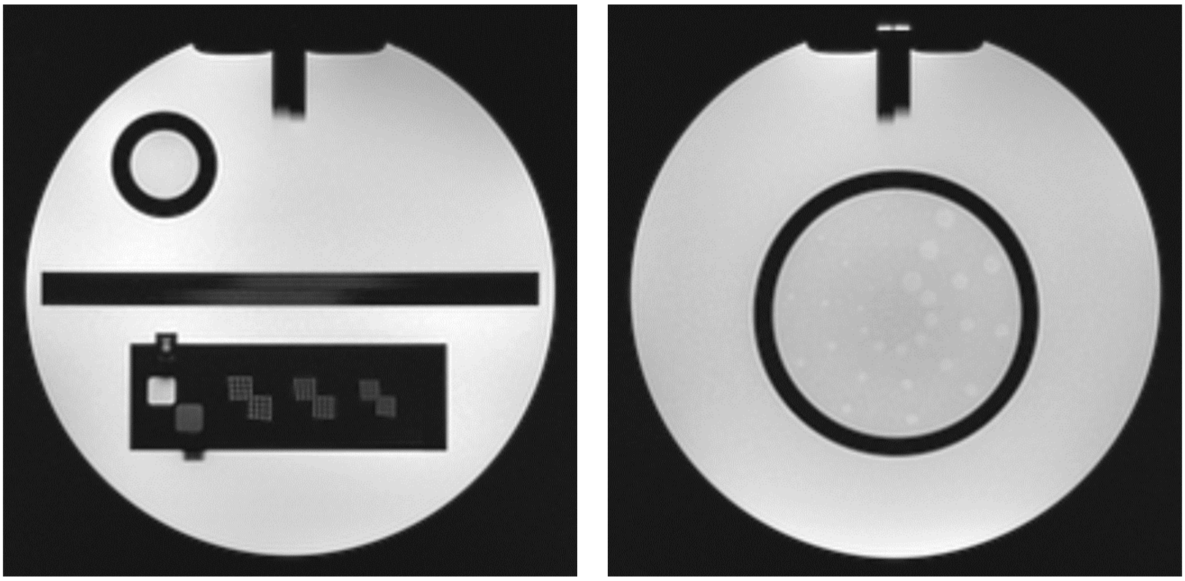

(a) Slice 1 (b) Slice 11

Figure S1: Example axial images of the American College of Radiologists phantom for the MR image quality test. Slices 1 (a) and 11 (b) are shown.

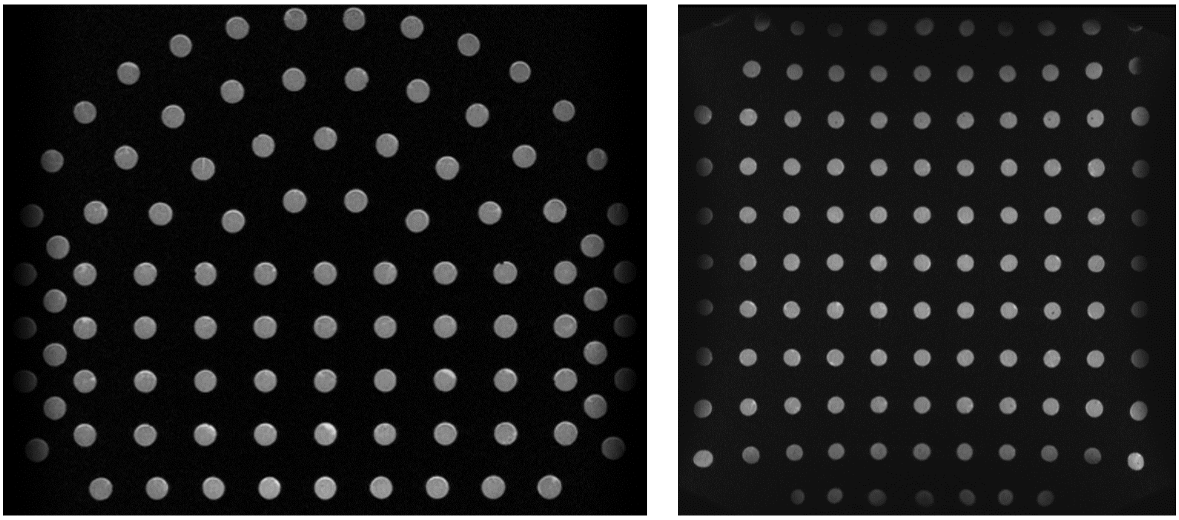

(a) Axial (b) Coronal

Figure S2: Example axial (a) and coronal (b) images of the GRADE phantom for the MR geometric accuracy test.

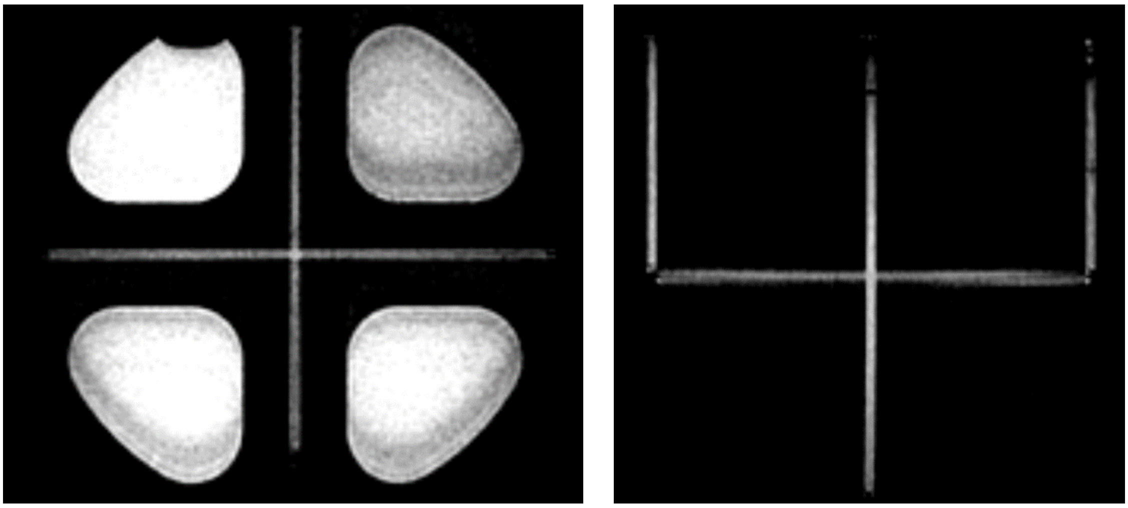

(a) Axial (b) Coronal

Figure S3: Example axial (a) and coronal (b) images of the Aquarius phantom for the mechanical accuracy test.

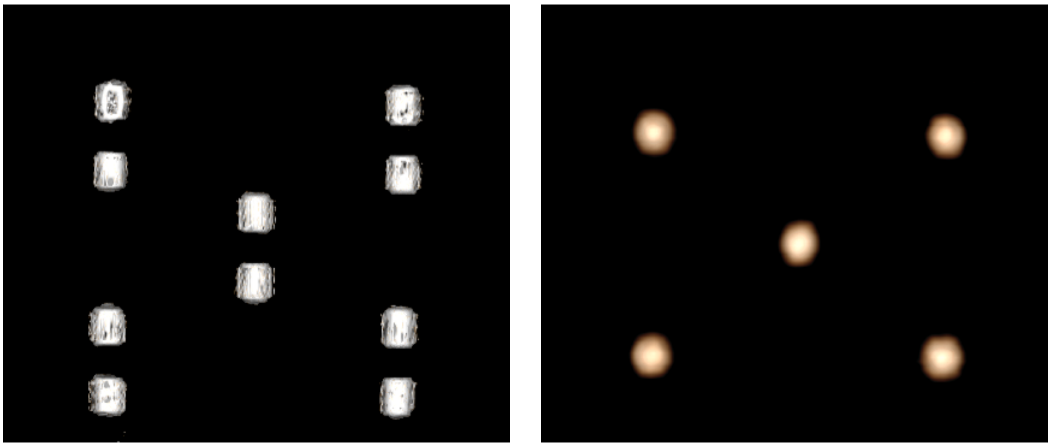

(a) MR (b) PET

Figure S4: Example MR (a) and PET (b) images of the VQC phantom for the PET-MR alignment test. Images are shown as 3D renderings as viewed from anterior to the phantom.

Supplementary Material B: Additional Information Methods

B.1 DW-MR Apparent Diffusion Coefficient Accuracy

The reference ADC values at 20°C were 1626×10^{-6} $mm^2\,s^{-1}$, 1009×10^{-6} $mm^2\,s^{-1}$ and 640×10^{-6} $mm^2\,s^{-1}$ for n-nonane, n-undecane and tridecane respectively.

B.2 PET Standard Uptake Value Accuracy

Three repeatability scans of the phantom were acquired on the same day, with the activty modified between scans 1 & 2 and 2 & 3. The initial activity in the phantom was 31.9 MBq at 13:45, measured with dose calibrator (CRC-15 PET, Capintec, New Jersey, USA). After scan 1, a small amount of FDG was then removed from the phantom into a syringe, an additional 8.9 MBq of FDG injected to the phantom and then as much as possible of the removed FDG returned.

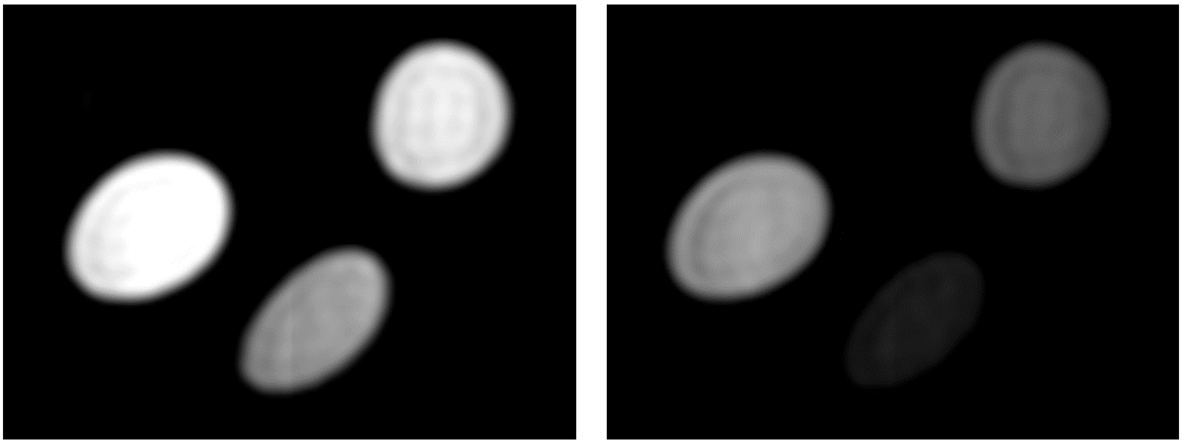

(a) $b = 50\,\mathrm{s\,mm^{-2}}$ (b) $b = 800\,\mathrm{s\,mm^{-2}}$

Figure S5: Example coronal images of the in-house DW-MR phanto for the ADC accuracy test. Images are shown for the $b = 50$ s mm^{-2} (a) and $b = 800$ s mm^{-2} (b).

The residual activity of both syringes was measured and subtracted from the decay-corrected initial activity to determine the total activity in the phantom for the second repeat measurement, which was 34.5 MBq at 14:20. The process was repeated for scan 3 with 10.7 MBq of FDG added, giving a total activity at 14:57 of 38.0 MBq.

Attenuation correction of the phantom was carried out using a CT of the phantom. A non-attenuation corrected PET image of the phantom was acquired and rigidly registered to the CT. The CT was resampled onto the PET image matrix in MICE Toolkit. The resampled CT and non-attenuation corrected PET were uploaded onto the scanner PET phantom library. On acquiring a PET scan of the phantom, the scanner automatically rigidly registered the acquired PET image to the phantom library PET image. This registration matrix was then used to align the phantom library CT to the newly acquired image, the CT was converted to 511 keV attenuation coefficients and then combined with an attenuation coefficient map of the spine coil and PET-MR couch. The combined attenuation correction map was used for the final PET image reconstruction (see figure S6).

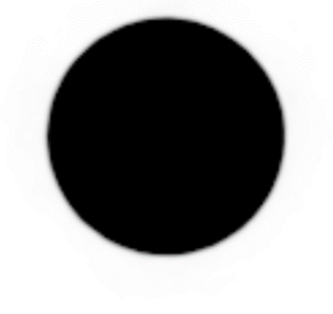

(a) PET axial

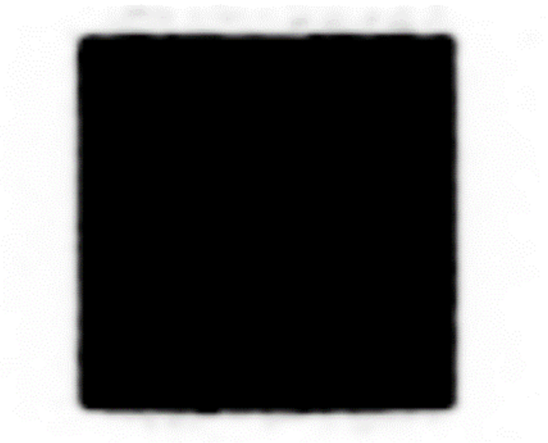

(b) PET coronal

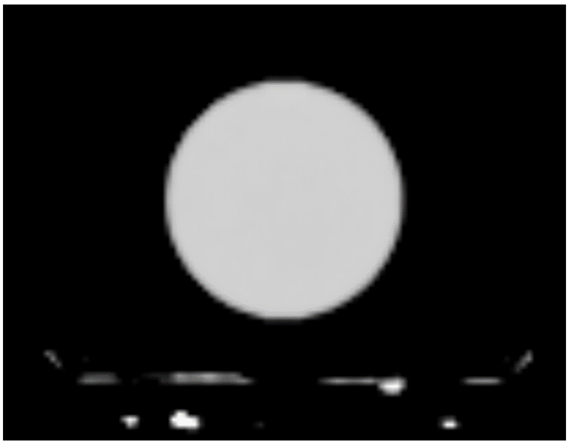

(c) AC map axial

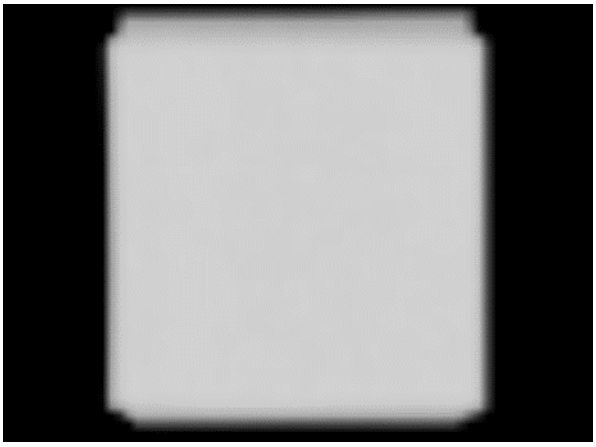

(d) AC map coronal

Figure S6: Example PET axial (a) and coronal (b) images of the uniform flood phantom for the PET SUV accuracy test. Also shown are the corresponding Attenuation Correction (AC) maps, (c) and (d), of the phantom. The axial AC map shows the PET couch and spine coil elements that were included in the AC map.
